# Meyerson-Nävus oder Melanom?

**DOI:** 10.1007/s00105-024-05447-z

**Published:** 2024-12-11

**Authors:** Julian Kühnl, Eva Schadelbauer, Rainer Hofmann-Wellenhof

**Affiliations:** Univ. Klinik für Dermatologie und Venerologie, Medizinische Universität Graz, Auenbruggerplatz 8, 8010 Graz, Österreich

## Anamnese

Ein 18-jähriger Patient stellte sich mit einer juckenden Hautveränderung am lateralen Thoraxbereich vor. Er berichtete, dass sich das betroffene Muttermal im Laufe der Zeit verändert hatte und von starkem Juckreiz begleitet war. Zur Linderung kratzte der Patient gelegentlich an dem Muttermal und behandelte es mit einer Wundsalbe. Abgesehen vom Juckreiz zeigte der Patient keine weiteren Symptome. In der Vorgeschichte war ein vollständig entferntes Melanom (Tumordicke unter 0,5 mm) am Nacken zu verzeichnen. Darüber hinaus bestanden keine relevanten Vorerkrankungen.

## Klinischer und dermatoskopischer Befund

**Abb. 1:**
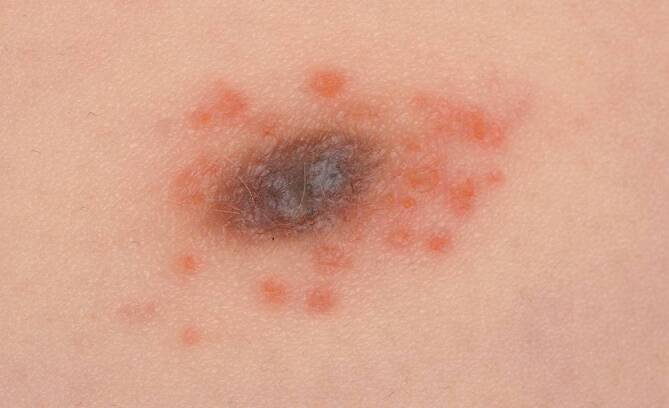
Klinische Aufnahme der beschriebenen Läsion mit deutlich sichtbarer entzündlicher Reaktion

Bei der klinischen Untersuchung zeigte sich links thorakal eine grau-bräunliche Plaque mit kleinen, erythematösen Papeln und vereinzelten Bläschen in der unmittelbaren Umgebung (Abb. 1). Die Plaque war gut umschrieben und wies keine Ulzeration oder Blutung auf. Es waren keine weiteren auffälligen Hautläsionen am Patienten erkennbar.

**Abb. 2:**
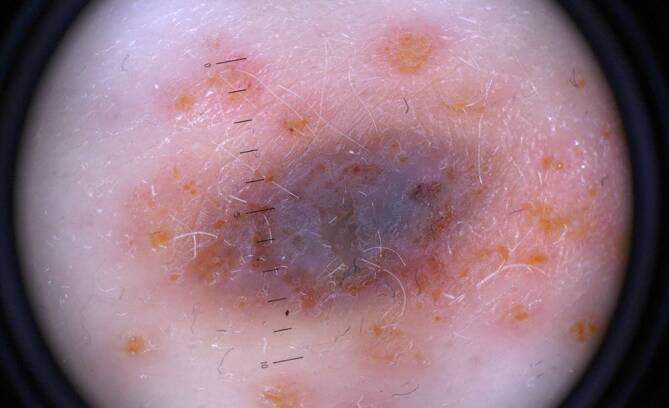
Dermatoskopische Aufnahme, besonders beachtenswert sind die Bläschen und Krusten in der direkten Umgebung der Läsion

Die dermatoskopische Untersuchung zeigte einen grau-blauen Schleier über der gesamten Plaque (Abb. 2) ohne erkennbares Netzwerk oder Schollen. Des Weiteren war die Plaque unscharf abgegrenzt. In der unmittelbaren Umgebung waren entzündliche Papeln und seröse Krusten zu sehen. Aufgrund des unklaren klinischen und dermatoskopischen Befundes wurde eine Totalexzision durchgeführt.

## Wie lautet Ihre Diagnose?

## Histologie

Das histologische Präparat zeigte einen melanozytären Nävus vom Compound-Typ. Fokale Erosionen waren von nekrotischen Keratinozyten begleitet, die teilweise eine ballonierende Degeneration aufwiesen. In den nekrotischen Bereichen fanden sich neutrophile Granulozyten. Eine gemischtzellige Entzündungsreaktion mit eosinophilen Granulozyten rundete den histologischen Befund ab. Basierend auf diesen Befunden, wurde die Diagnose eines melanozytären Compound-Nävus mit einer Herpes-simplex-Infektion gestellt [2].

## Diskussion

**Abb. 3:**
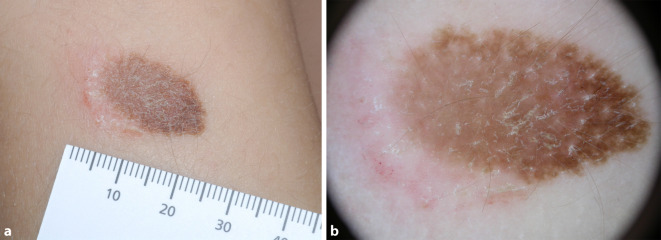
Ein Meyerson-Nävus zum Vergleich. **a** Deutlich erkennbar ist der gerötete, leicht schuppende Randsaum mit vereinzelten Bläschen. Der Nävus im Zentrum zeigt ein retikuläres Muster (**b**)

Für den vorliegenden Fall wurden mehrere Differenzialdiagnosen in Betracht gezogen. Ein Meyerson-Nävus war die ursprüngliche klinische Diagnose. Dafür sprach die Tatsache, dass der Patient neben der melanozytären Proliferation eine auffällige perifokale Entzündungsreaktion aufwies. Darüber hinaus kann das Auftreten eines grau-blauen Schleiers und einer Regression, wie in der Dermatoskopie beobachtet, mit einem Meyerson-Nävus vereinbar sein, wie die Abb. 3 zeigt [1, 3].

Ein Meyerson-Nävus ist eine entzündliche Reaktion um ein Muttermal herum, die oft durch Ekzeme oder andere dermatitische Prozesse ausgelöst wird. Diese Erscheinung ist durch die Depigmentierung um den Nävus herum gekennzeichnet. Allerdings sprach gegen die Diagnose eines Meyerson-Nävus die mikroskopische Untersuchung, die eine melanozytäre Proliferation vom Compound-Typ und die typischen Merkmale einer Herpesinfektion zeigte. Weitere Differenzialdiagnosen umfassten ein atypisches Melanom, eine Kollisionsläsion oder eine kontaktallergische Reaktion.

**Diagnose:** melanozytärer Compound-Nävus mit begleitender Herpes-simplex-Infektion

Zusammenfassend lässt sich feststellen, dass es sich bei dem vorliegenden Fall um einen melanozytären Compound-Nävus mit einer begleitenden Herpes-simplex-Infektion handelte. Diese ungewöhnliche Kombination unterstreicht die Wichtigkeit einer sorgfältigen klinischen, dermatoskopischen und histologischen Untersuchung bei atypischen Hautveränderungen.
